# *Ab initio* calculation of valley splitting in monolayer *δ*-doped phosphorus in silicon

**DOI:** 10.1186/1556-276X-8-111

**Published:** 2013-02-27

**Authors:** Daniel W Drumm, Akin Budi, Manolo C Per, Salvy P Russo, Lloyd C L Hollenberg

**Affiliations:** 1 School of Physics, The University of Melbourne, Parkville, Victoria 3010, Australia; 2 School of Applied Sciences, RMIT University, Melbourne, Victoria 3001, Australia; 3 Virtual Nanoscience Laboratory, CSIRO Materials Science and Engineering, Parkville, Victoria 3052, Australia

**Keywords:** Density functional theory, Valley splitting, *δ*-Doped layers, Phosphorus in silicon, Basis sets, 73.22.-f, 31.15.ae, 71.15.Mb

## Abstract

The differences in energy between electronic bands due to valley splitting are of paramount importance in interpreting transport spectroscopy experiments on state-of-the-art quantum devices defined by scanning tunnelling microscope lithography. Using vasp, we develop a plane-wave density functional theory description of systems which is size limited due to computational tractability. Nonetheless, we provide valuable data for the benchmarking of empirical modelling techniques more capable of extending this discussion to confined disordered systems or actual devices. We then develop a less resource-intensive alternative via localised basis functions in siesta, retaining the physics of the plane-wave description, and extend this model beyond the capability of plane-wave methods to determine the *ab initio* valley splitting of well-isolated *δ*-layers. In obtaining an agreement between plane-wave and localised methods, we show that valley splitting has been overestimated in previous *ab initio* calculations by more than 50%.

## Background

The study of the quantum properties of low-dimensional and doped structures is central to many nanotechnology applications [1-15]. Quantum devices in silicon have been the subject of concentrated recent interest, both experimental and theoretical, including the recent discussion of Ohm’s law at the nanoscale [16]. Efforts to make such devices have led to atomically precise fabrication methods which incorporate phosphorus atoms in a single monolayer of a silicon crystal [17-20]. These dopant atoms can be arranged into arrays [21] or geometric patterns for wires [16,22] and associated tunnel junctions [23], gates, and quantum dots [24,25] - all of which are necessary components of a functioning device [26]. The patterns themselves define atomically abrupt regions of doped and undoped silicon. While silicon, bulk-doped silicon, and the physics of the phosphorus incorporation [27] are well understood, models of this quasi-two-dimensional phosphorus sheet are still in their initial stages. In particular, it is critical in many applications to understand the effect of this confinement on the conduction band valley degeneracy, inherent in the band structure of silicon. For example, the degeneracy of the valleys has the potential to cause decoherence in a spin-based quantum computer [28,29], and the degree of valley degeneracy lifting (valley splitting) defines the conduction properties of highly confined planar quantum dots [26].

The importance of understanding valley splitting in monolayer *δ*-doped Si:P structures has led to a number of theoretical works in recent years, spanning several techniques, from pseudo-potential theories via planar Wannier orbital bases [30], density functional theory (DFT) via linear combination of atomic orbital (LCAO) bases [31,32], to tight-binding models [33-37] and effective mass theories (EMT) [38-40]. We note that several of these papers are based upon the assumption that the effective masses of *δ*-doped P in Si remain unchanged from bulk-doped values [38,39], an assumption which has been challenged [30,33]. Others assume doping over a multi-atomic plane band [33,38] which no longer represents the state of the art in fabrication. There is currently little agreement between the valley splitting values obtained using these methods, with predictions ranging between 5 to 270 meV, depending on the calculational approach and the arrangement of dopant atoms within the *δ*-layer. Density functional theory has been shown to be a useful tool in predicting how quantum confinement or doping perturbs the bulk electronic structure in silicon- and diamond-like structures [41-45]. The work of Carter et al. [31] represents the first attempt using DFT to model these devices by considering explicitly doped *δ*-layers, using a localised basis set and the assumption that a basis set sufficient to describe bulk silicon will also adequately describe P-doped Si. It might be expected, therefore, that the removal of the basis set assumption will lead to the best *ab initio* estimate of the valley splitting available, for a given arrangement of atoms. In the context of describing experimental devices, it is important to separate the effects of methodological choices, such as this, from more complicated effects due to physical realities, including disorder.

In this paper, we determine a consistent value of the valley splitting in explicitly *δ*-doped structures by obtaining convergence between distinct DFT approaches in terms of basis set and system sizes. We perform a comparison of DFT techniques, involving localised numerical atomic orbitals and delocalised plane-wave (PW) basis sets. Convergence of results with regard to the amount of Si ‘cladding’ about the *δ*-doped plane is studied. This corresponds to the normal criterion of supercell size, where periodic boundary conditions may introduce artificial interactions between replicated dopants in neighbouring cells. A benchmark is set via the delocalised basis for DFT models of *δ*-doped Si:P against which the localised basis techniques are assessed. Implications for the type of modelling being undertaken are discussed, and the models extended beyond those tractable with plane-wave techniques. Using these calculations, we obtain converged values for properties such as band structures, energy levels, valley splitting, electronic densities of state and charge densities near the *δ*-doped layer.

The paper is organised as follows: the ‘Methods’ section outlines the parameters used in our particular calculations; we present the results of our calculations in the ‘Results and discussion’ section and draw conclusions in the ‘Conclusions’ section. An elucidation of effects modifying the bulk band structure follows in Appendices 1 and 2 to provide a clear contrast to the properties deriving from the *δ*-doping of the silicon discussed in the paper. The origin of valley splitting is discussed in Appendix 3.

## Methods

Density functional theory calculations have been carried out using both plane-wave and LCAO basis sets. For the PW basis set, the Vienna *ab initio* simulation package (vasp) [46] software was used with projector augmented wave [46,47] pseudo-potentials for Si and P. Due to the nature of the PW basis set, there exists a simple relationship between the cut-off energy and basis set completeness. For the structures considered in this work, the calculations were found to be converged for PW cut-offs of 450 eV.

Localised basis set calculations were performed using the Spanish Initiative for Electronic Simulations with Thousands of Atoms (siesta) [48] software. In this case, the P and Si ionic cores were represented by norm-conserving Troullier-Martins pseudo-potentials [49]. The Kohn-Sham orbitals were expanded in the default single-*ζ* polarized (SZP) or double-*ζ *polarized (DZP) basis sets, which consist of 9 and 13 basis functions per atom, respectively. Both the SZP and DZP sets contain *s*-, *p*-, and *d*-type functions. These calculations were found to be converged for a mesh grid energy cut-off of 300 Ry. In all cases, the generalized gradient approximation PBE [50] exchange-correlation functional was used.

The lattice parameter for bulk Si was calculated using an eight-atom cell and found to be converged for all methods with a 12 × 12 × 12 Monkhorst-Pack (MP) *k*-point mesh [51]. The resulting values are presented in Table 1 and were used in all subsequent calculations.

**Table 1:** Eight-atom cubic unit cell equilibrium lattice parameters for different methods used in this work

**Method**	***a***_**0**_** (Å)**
PW (vasp)	5.469
DZP (siesta)	5.495
SZP (siesta)	5.580

**Figure 1:**
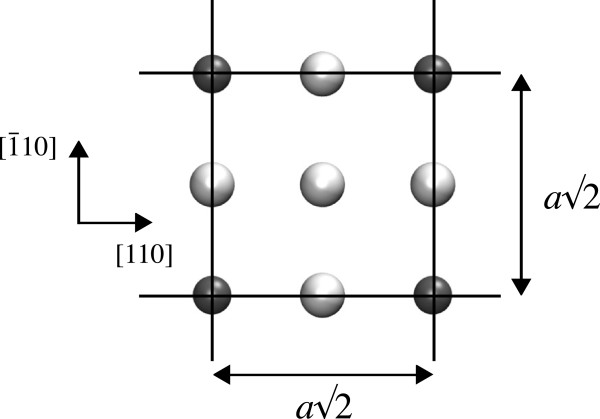
**(001) Planar slice of the *c*(2*×*2) structure at the 1/4 ML doped monolayer.** One of the Si sites has been replaced by a P atom (shown in dark gray). The periodic boundaries are shown in black.

In modelling *δ*-doped Si:P, as used in another work [26], we adopted a tetragonal supercell description of the system, akin to those of other works [30,31]. In accordance with the experiment, we inserted the P layer in a monatomic (001) plane as one atom in four to achieve 25% doping. This will henceforth be referred to as 1/4 monolayer (ML) doping. In this case, the smallest repeating in-plane unit had 4 atoms/ML (to achieve one in four dopings) and was a square with the sides parallel to the [110] and $\bar{1}$10] directions. The square had a side length $a\sqrt{2}$ (see Figure 1), where *a* is the simple cubic lattice constant of bulk silicon. The phosphorus layers had to be separated by a considerable amount of silicon due to the large Bohr radius of the hydrogen-like orbital introduced by P in Si (approximately 2.5 nm). Carter et al. [31] showed that this far exceeded the sub-nanometre cell side length. If desired, cells with a lower in-plane density of dopants may be constructed by lengthening the cell in the *x* and *y* directions, such that more Si atoms occupy the doped monolayer in the cell - though this would significantly increase the computational cost of such a calculation.

**Figure 2:**
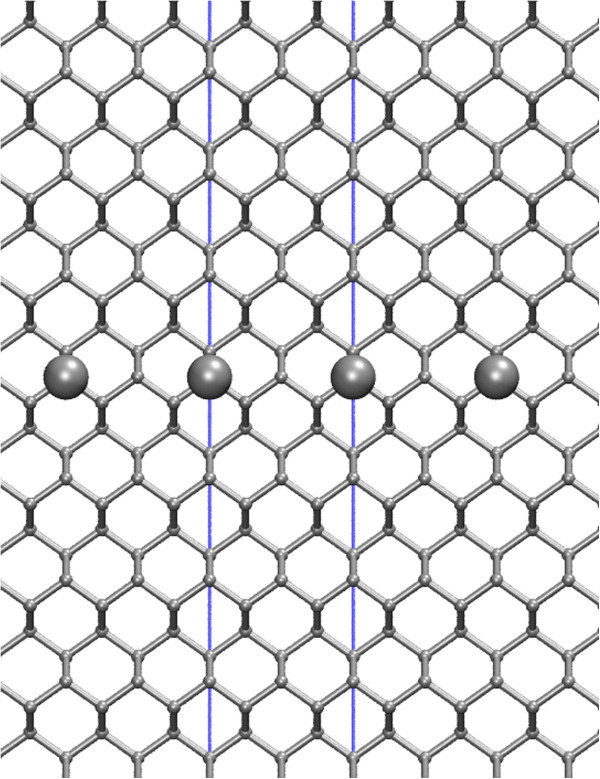
**Ball and stick model of a *δ*-doped Si:P layer viewed along the [110] direction.** Thirty-two layers in the [001] direction are shown. Si atoms (small gray spheres), P atoms (large dark gray spheres), covalent bonds (gray sticks), repeating cell boundary (solid line).

A collection of tetragonal cells comprising 4, 8, 16, 32, 40, 60, 80, 120, 160 and 200 monolayers was constructed, having four atomic sites per monolayer and oriented with faces in the [110], [ $\bar{1}$10], and [001] directions (see Figure 2). Cells used in PW calculations began at 4 layers and ran to 80 layers; larger cells were not computationally tractable with this method. SZP and DZP models began at 40 layers to overlap with PW for the converging region and were then extended to their tractable limit (200 and 160 layers, respectively) to study convergence past the capability of PW.

For tetragonal cells, the *k*-point sampling was set as a 9 × 9 ×* N *Γ-centred MP mesh as we have found that failing to include Γ in the mesh can lead to the anomalous placement of the Fermi level on band structure diagrams. *N* varied from 12 to 1 as the cells became more elongated (see Appendix 1). We note that, as mentioned in the work of Carter et al. [32], the large supercells involved required very gradual (<0.1%) mixing of the new density matrix with the prior step, leading to many hundreds of self-consistent cycles before convergence was achieved.

Although it has been previously found that relaxing the positions of the nuclei gave negligible differences (<0.005 Å) to the geometry [31], this was for a 12-layer cell and may not have included enough space between periodic repetitions of the doping plane for the full effect to be seen. Whilst a 40-layer model was optimised in the work of Carter et al. [32], this made use of a mixed atom pseudo-potential and is not explicitly comparable to the models presented here. We have performed a test relaxation on a 40-layer cell using the PW basis (vasp). The maximum subsequent ionic displacement was 0.05 Å, with most being an order of magnitude smaller. The energy gained in relaxing the cell was less than 37 meV (or 230 *μ*eV/atom). We therefore regarded any changes to the structure as negligibly small, confirming the results of Carter et al. [31,32], and proceeded without ionic relaxation.

Single-point energy calculations were carried out with both software programs; for vasp, the electronic energy convergence criterion was set to 10^−6^eV, and the tetrahedron method with Blöchl correction [52] was used. For siesta, a two-stage process was carried out: Fermi-Dirac electronic smearing of 300 K was applied in order to converge the density matrix within a tolerance of one part in 10^−4^; the calculation was then restarted with the smearing of 0 K, and a new electronic energy tolerance criterion of 10^−6^ eV was applied (except for the 120- and 160-layer DZP models for which this was intractable; a tolerance of 10^−4^ eV was used in these cases). This two-stage process aided convergence as well as ensuring that the energy levels obtained were comparably accurate across methods. In addition, for each doped cell thus developed and studied, an undoped bulk Si cell of the same dimensions was constructed to aid in isolating those features primarily due to the doping.

## Results and discussion

### Analysis of band structure

Once converged charge densities were obtained, band structures were calculated along the *M*–Γ–*X* high-symmetry pathway (as shown in Appendix 1), using at least 20 *k*-points between high-symmetry points. For comparative purposes, the band structures have all been aligned at the valence band maximum (VBM).

**Figure 3:**
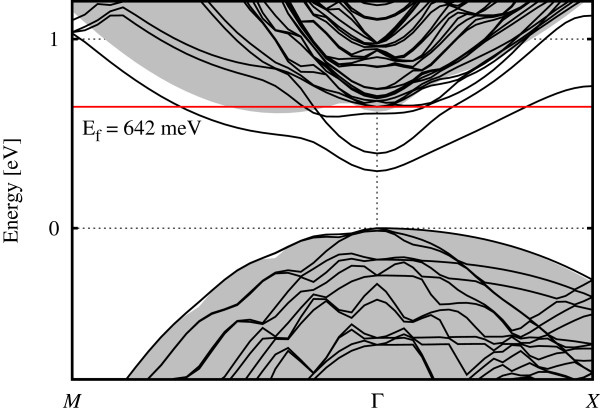
**Full band structure (colour online) of the 40-layer tetragonal system calculated using PW (vasp).** Bulk band structure (shaded gray background), doped band structure (solid black) and Fermi level (labelled solid red).

Figure 3 contrasts the bulk and doped band structures for the 40-layer PW calculation. DZP and SZP results are qualitatively similar on this scale, albeit with different band energies in the SZP model, and are omitted in the interest of clarity in the diagram. As discussed in Appendix 2, it is evident from the bulk values that the elongated cells have led to the folding of two conduction band minimum valleys towards the Γ point. Also visible is the difference that the doping potential makes to the system; what was the lowest unoccupied orbital (Γ_1_ band) in the bulk is now dragged down in energy by the extra ionic potential. It is of note that the region near Γ, corresponding to the *k*_*z *_valleys which can be modelled as having different effective masses to the *k*_*x*,*y *_valleys, [30] is brought lower than the region corresponding to the *k*_*x*,*y*_ valleys and is non-degenerate. The second (Γ_2_) band behaves in a similar fashion. The third (*δ*) band appears to maintain a minimum away from the Γ point in the Σ_TET_ direction (which is equivalent to the Δ_FCC _direction; see Appendix 1) but in a less parabolic fashion than the lower two; its minimum is similar to the value at Γ. This band is non-degenerate along this particular direction in *k*-space, but due to the supercell symmetry, it is actually fourfold degenerate, in contrast to the other bands.

The Fermi level for the doped system is also shown, clearly being crossed by all three of these bands which are therefore able to act as open channels for conduction.

**Figure 4:**
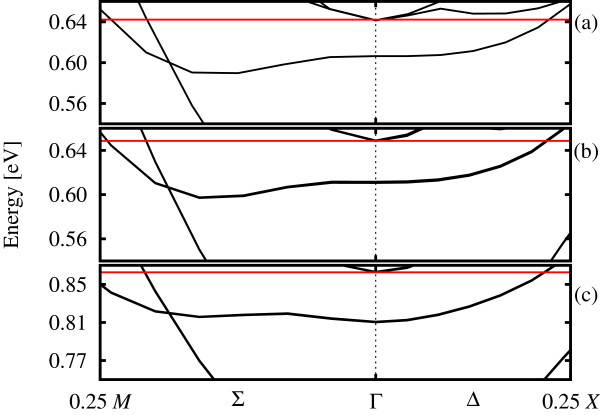
**Band structure (colour online) of the 40-layer tetragonal system zoomed in on the *δ *band.** (**a**) PW (vasp), (**b**) DZP (siesta) and (**c**) SZP basis sets were used. Fermi level is shown by a solid horizontal red line.

As mentioned above, the band structures are similar across all methods, but upon detailed inspection, important differences come to light. A closer look at the *δ *band shows a qualitative difference between the predictions using SZP (Figure 4c) and the PW and DZP results (Figure 4a,b): the models with a more complete basis predict the band minimum to occur in the Σ_TET_(Δ_FCC_) direction, below the value at Γ, while the SZP band structure shows the reverse - the minimum at Γ, a similar amount below a secondary minimum in the Σ_TET _direction, a qualitative difference.

**Figure 5:**
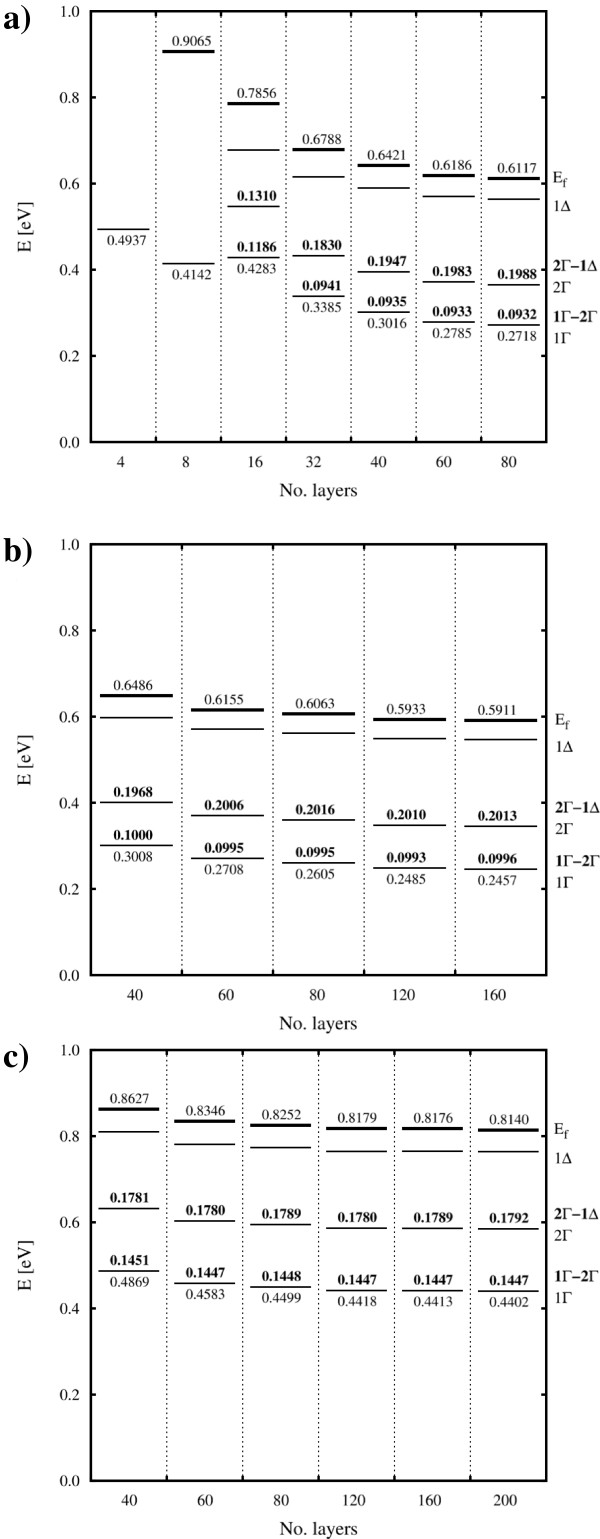
**Minimum band energies for tetragonal systems with 1/4 ML doping.** (**a**) PW (vasp), (**b**) DZP (siesta) and (**c**) SZP (siesta) basis sets were used. Fermi level also shown where appropriate. Bold numbers indicate energy differences between band minima.

The difference between the energies of the first two band minima (Γ_1_− Γ_2_, illustrated in Figure 5), or the valley splitting, from the PW and DZP calculations, agrees with each other to within ∼6 meV. Significantly, the value obtained using our SZP basis set differs by 52 meV, some 55% larger than the value obtained using the PW basis set. The importance of this discrepancy cannot be overstated; valley splitting is directly relatable to experimentally observable resonances in transport spectroscopy of devices made with this *δ*-doping technology (see [26]).

In the smallest cells (<16 layers), less than three bands are observed. This is likely due to the lack of cladding in the *z* direction, leading to a significant interaction between the dopant layers, raising the energy of each band. Whilst the absolute energy of each level still varies somewhat, even with over 100 layers incorporated, we find that the Γ_1_–Γ_2 _values are well converged with 80 layers of cladding for all methods (see Figure 5). Indeed, they may be considered reasonably converged even at the 40-layer level (0.5 meV or less difference to the largest models considered). The differences between the energies of the second and third band minima (Γ_2_–*δ *splittings) are also shown in Figure 5 and show good convergence (within 1 meV) for cells of 80 layers or larger.

The Fermi level follows a similar pattern to the Γ- and *δ*-levels. In particular, the gap between the Fermi level and Γ_1 _level does not change by more than 1 meV from 60 to 160 layers.

Given that the properties of interest are the differences between the energy levels, rather than their absolute values (or position relative to the valence band), in the interest of computational efficiency, we observe that using the DZP basis with 80 layers of cladding is sufficient to achieve consistent, converged results.

### Valley splitting

Table 2 summarises the valley splitting values of 1/4 ML P-doped silicon obtained using different techniques, showing a large variation in the actual values. In order to make sense of these results, it is important to note two major factors that affect valley splitting: the doping method and the arrangement of phosphorus atoms in the *δ*-layer. As the results from the work of Carter et al. [32] show, the use of implicit doping causes the valley splitting value to be much smaller than in an explicit case (∼7 meV vs. 120 meV). It is also shown that the use of random P coverage on the *δ*-layer reduces the valley splitting value by only 40 to 50 meV compared with the fully ordered placement, leaving a large discrepancy between the valley splitting results from implicit and explicit doping. This large decrease in valley splitting due to implicit doping can be explained by the smearing of the doping layer in the direction normal to the *δ*-layer, thereby decreasing the quantum confinement effect responsible for breaking the degeneracy in the system. Carter et al. [32] also shows that the arrangement of the phosphorus atoms in the *δ*-layer strongly influences the valley splitting value. In particular, they showed that there is a difference of up to 220 meV between P doping along the [110] direction and along the [100] direction. It should be noted, however, that deterministic nearest-neighbour donor placements are not yet physically realisable due to the P incorporation mechanism currently employed [27,53]. Similarly, the perfectly ordered arrangement discussed here is highly improbable, given the experimental limitations, but represents the ideal case from which effects such as disorder can be studied.

**Table 2:** Valley splitting values of 1/4 ML P-doped silicon obtained using different techniques

**Technique**	**Number of**	**Valley**
	**layers**	**splitting**
		**(meV)**
Planar Wannier orbital^a^[30]	1,000	20
Tight binding (4 K)^b^[34]	∼150	∼17
Tight binding (4 K)^b^[37]	120	25
Tight binding (300 K)^b^[36]	∼150	∼17
	40	7
	80	6
DFT, SZP basis set ^a^[32]	120	6
	160	6
	200	6
DFT, SZP: ordered ^b^[31]	40	120
DFT, SZP: random disorder ^b^[31]	40	∼70
DFT, SZP: [110] direction alignment ^b^[32]	40	∼270
DFT, SZP: dimers ^b^[32]	40	∼85
DFT, SZP: random disorder ^b^[32]	40	∼80
DFT, SZP: clusters ^b^[32]	40	∼65
DFT, SZP: [100] direction alignment ^b^[32]	40	∼50
DFT, SZP: ordered, *M*=4^b,c^[32]	80	153
DFT, SZP: ordered, *M*=6^b,c^[32]	80	147
DFT, SZP: ordered, *M*=10^b,c^[32]	80	147
	40	145.1
	60	144.7
SZP, *M*=9 (this work)^b,c^	80	144.8
	120	144.7
	160	144.7
	200	144.7
	16	118.6
	32	94.1
PW, *M*=9 (this work)^b,d^	40	93.5
	60	93.3
	80	93.2
	40	100
	60	99.5
DZP, *M*=9 (this work)^b,c^	80	99.5
	120	99.3
	160	99.6

Techniques are grouped by similarity. ^a^Implicit doping; ^b^Explicit doping; ^c^*M *×* M *× 1*k*-points; ^d^*M *×* M *×* Nk*-points; *N* as in Appendix 1.

Our results show that valley splitting is highly sensitive to the choice of basis set. Due to the nature of PW basis set, it is straightforward to improve its completeness by increasing the plane-wave cut-off energy. In this way, we establish the most accurate valley splitting value within the context of density functional theory. Using this benchmark value, we can then establish the validity and accuracy of other basis sets, which can be used to extend the system sizes to that beyond what is practical using a PW basis set. As seen in Table 2, the valley splitting value converges to 93 meV using 80-layer cladding. The DZP localised basis set gives an excellent agreement at 99.5 meV using 80-layer cladding (representing a 7% difference). On the other hand, our SZP localised basis set gave a value of 145 meV using the same amount of cladding. This represents a significant difference of 55% over the value obtained using PW basis set and demonstrates that SZP basis sets are unsuitable for accurate determination of valley splitting in these systems.

### Density of states

**Figure 6:**
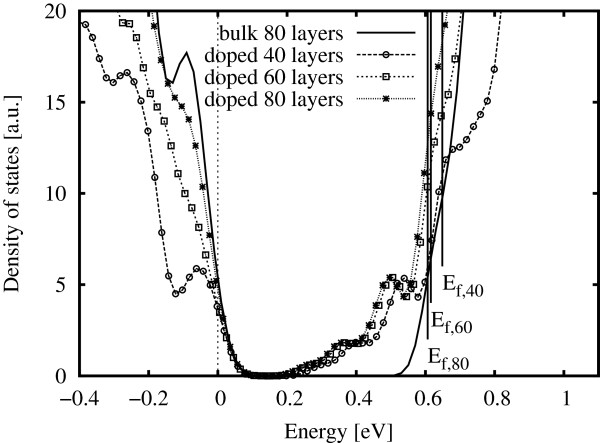
**Electronic densities of states for tetragonal systems with 0 and 1/4 ML doping.** The DZP (siesta) basis set was used. The Fermi level is indicated by a solid vertical line with label, and 50-meV smearing was applied for visualization purposes.

The electronic density of states (eDOS) was calculated for each cell. Figure 6 compares the unscaled eDOS for bulk 80-layer cells to that of doped cells varying from 40 to 80 layers. The bulk bandgap is visible, with the conduction band rising sharply to the right of the figure. The doped eDOS exhibits density in the bulk bandgap, although the features of the spectra differ slightly according to the basis set used.

The Fermi energy exhibits convergence with respect to the amount of cladding, as reported above. It is also notable that the eDOS within the bandgap are nearly identical regardless of the cell length (in *z*). This indicates that layer-layer interactions are negligibly affecting the occupied states and, therefore, that the applied ‘cladding’ is sufficient to insulate against these effects.

### Electronic width of the plane

**Figure 7:**
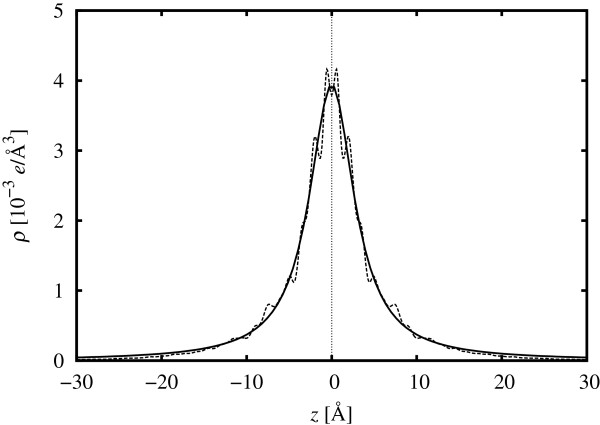
**Planar average of donor-electron density as a function of *z*-position for 1/4 ML-doped 80-layer cell.** The DZP basis set was used. The fitted Lorentzian function is also shown.

In order to quantify the extent of the donor-electron distribution, we have integrated the local density of states between the VBM and Fermi level and have taken the planar average with respect to the *z*-position. Figure 7 shows the planar average of the donor electrons (a sum of both spin-up and spin-down channels) for the 80-layer cell calculated using the DZP basis set. After removing the small oscillations related to the crystal lattice to focus on the physics of the *δ*-layer, by Fourier transforming, a Lorentzian function was fitted to the distribution profile. (Initially, a three-parameter Gaussian fit similar to that used in [40] was tested, but the Lorentzian gave a better fit to the curve.)

Table 3 summarises the maximum donor-electron density and the full width at half maximum (FWHM) for the 1/4 ML-doped cells, each calculated from the Lorentzian fit. Both of these properties are remarkably consistent with respect to the number of layers, indicating that they have converged sufficiently even at 40 layers.

**Table 3:** **Calculated maximum donor-electron density,*ρ***_**max**_**, and FWHM**

**Number of**	***ρ***_**max**_	**FWHM**
**layers**	**(×10**^**−3**^***e*/Å)**	**(Å)**
40	3.8	6.2
60	3.9	6.2
80	3.9	6.5

Values are presented as a function of the number of layers in 1/4 ML-doped cells. The DZP basis set was used.

Our results differ from a previous DFT calculation [32] which cited an FWHM of 5.62 Å for a 1/4 ML-doped, 80-layer cell calculated using the SZP basis set (and 10 × 10 × 1 *k*-points). We note that those values were taken from the unfitted, untransformed donor-electron distribution and represent an approximately 15% underestimation in comparison with the DZP result. The peak height is not shown in the work of Carter et al. [32], but the value from another work [31] (1.7 × 10^21^ e/cm^3^) is a factor of 0.44 smaller than the peak we observe here. This may be due, to some extent, to the larger width of the SZP model leading to an associated lowering of the peak density.

## Conclusions

In this article, we have studied the valley splitting of the monolayer *δ*-doped Si:P, using a density functional theory model with a plane-wave basis to establish firm grounds for comparison with less computationally intensive localised-basis *ab initio* methods. We found that the description of these systems (by density functional theory, using SZP basis functions) overestimates the valley splitting by over 50%. We show that DZP basis sets are complete enough to deliver values within 10% of the plane-wave values and, due to their localised nature, are capable of calculating the properties of models twice as large as is tractable with plane-wave methods. These DZP models are converged with respect to size well before their tractable limit, which approaches that of SZP models.

Valley splittings are important in interpreting transport spectroscopy experiment data, where they relate to families of resonances, and in benchmarking other theoretical techniques more capable of actual device modelling. It is therefore pleasing to have an *ab initio* description of this effect which is fully converged with respect to basis completeness as well as the usual size effects and *k*-point mesh density.

We have also studied the band structures with all three methods, finding that the DZP correctly determines the *δ*-band minima away from the Γ point, where the SZP method does not. We show that these minima occur in the Σ direction for the type of cell considered, not the *δ *direction as has been previously reported. Having established the DZP methodology as sufficient to describe the physics of these systems, we then calculated the electronic density of states and the electronic width of the *δ*-layer. We found that previous SZP descriptions of these layers underestimate the width of the layers by almost 15%.

We have shown that the properties of interest of *δ*-doped Si:P are well converged for 40-layer supercells using a DZP description of the electronic density. We recommend the use of this amount of surrounding silicon, and technique, in any future DFT studies of these and similar systems - especially if inter-layer interactions are to be minimised.

## Appendix 1

### Subtleties of bandstructure

Regardless of the type of calculation being undertaken, a band structure diagram is inherently linked to the type (shape and size) of cell being used to represent the system under consideration. For each of the 14 Bravais lattices available for three-dimensional supercells, a particular Brillouin zone (BZ) with its own set of high-symmetry points exists in reciprocal space [54]. Similarly, each BZ has its own set of high-symmetry directions. Some of these BZs share a few high-symmetry point labels (or directions), such as *X* or *L* (*δ* or Σ), and they all contain Γ, but these points are not always located in the same place in reciprocal space.

A simple effect of this can be seen by increasing the size of a supercell. This has the result of shrinking the BZ and the coordinates of high-symmetry points on its boundary by a corresponding factor. Consider the conduction band minimum (CBM) found at the *δ* valley in the Si conduction band. This is commonly located at $k_{0}∼0.85\frac{2\Pi }{a}$ in the *δ* direction towards *X* (also *Y*, *Z* and their opposite directions). Should we increase the cell by a factor of 2, the BZ will shrink (BZ→BZ’), placing the valley outside the new BZ boundary (past *X*’); however, a valid solution in any BZ must be a solution in all BZs. This results in the phenomenon of band folding, whereby a band continuing past a BZ boundary reenters the BZ on the opposite side. Since the *X* direction in a face-centred cubic (FCC) BZ is sixfold symmetric, a solution near the opposite BZ boundary is also a solution near the one we are focussing on. This results in the appearance that the band continuing past the BZ boundary is ‘reflected’, or folded, back on itself into the first BZ. Since the new BZ boundary in this direction is now at $k_{\text{BZ}}^{{}^{\prime }}=X^{{}^{\prime }}=0.5\frac{2\Pi }{a}$, the location of the valley will be at $k_{0}^{{}^{\prime }}=X^{{}^{\prime }}-\left(k_{0}-X^{{}^{\prime }}\right)∼0.15\frac{2\Pi }{a}$, as mentioned in the work of Carter et al. [31]. Each further increase in the size of the supercell will result in more folding (and a denser band structure). Care is therefore required to distinguish between a new band and one which has been folded due to this effect when interpreting band structure.

**Figure 8:**
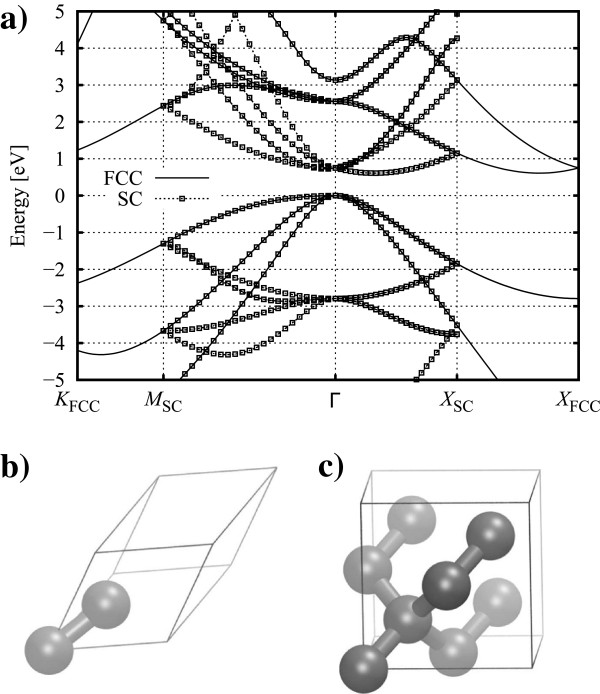
**Band structure and physical structure of FCC and SC cells.** (**a**) Typical band structure of bulk Si for two-atom FCC (solid lines) and eight-atom SC cells (dotted lines with squares), calculated using the vasp plane-wave method (see ‘Methods’ section). (**b**) Two-atom FCC cell. (**c**) Eight-atom SC cell.

Continuing with our example of silicon, whilst the classic band structure [55] is derived from the bulk Si primitive FCC cell (containing two atoms), it is often more convenient to use a simple cubic (SC) supercell (eight atoms) aligned with the 〈100〉 crystallographic directions. In this case, we experience some of the common labelling; the *δ* direction is defined in the same manner for both BZs, although we see band folding (in a similar manner to that discussed previously) due to the size difference of the reciprocal cells (see Figure 8). We also see a difference in that, although the Σ direction is consistent, the points at the BZ boundaries have different symmetries and, therefore, label (*K*_FCC_, *M*_SC_). (The *L*_FCC_ point and ⋀ _FCC _direction have no equivalent for tetragonal cells, and hence, we do not consider band structure in that direction here).

**Figure 9:**
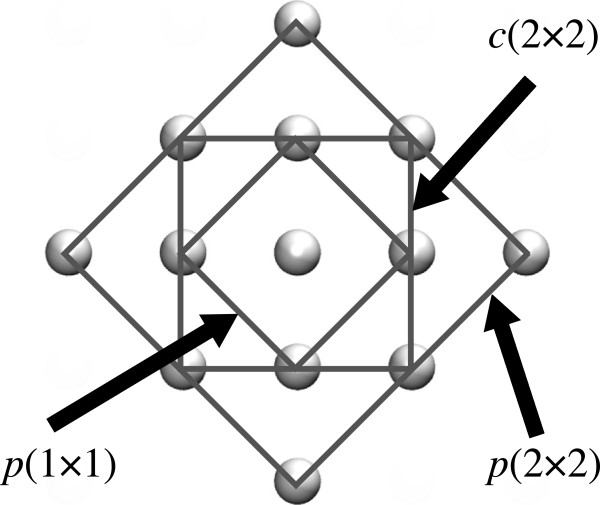
**Geometrical difference between the simple cubic and tetragonal cells.** A (001) planar cut through an atomic monolayer is shown.

**Figure 10:**
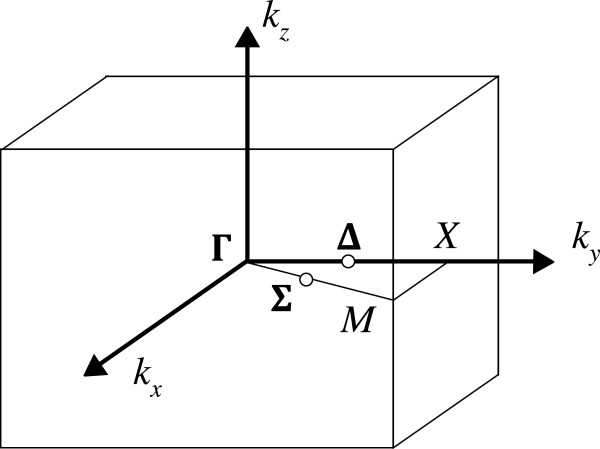
**The Brillouin zone for a tetragonal cell.** The *M*–Γ–*X* path used in this work is shown.

**Figure 11:**
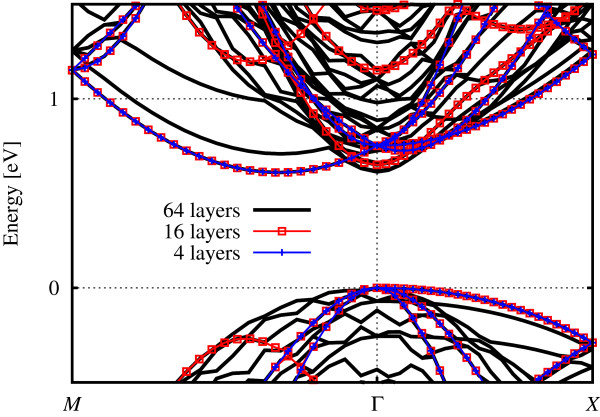
**Band structure (colour online) diagram for tetragonal bulk Si structures with increasing number of layers.** The vasp plane wave method was used (see ‘Methods’ section).

Consider now the *δ*-doping case discussed in the ‘Methods’ section, where we wish to align our cell with the [110] and [ $\bar{1}$10] directions (by rotating the cell 45° anticlockwise about *z*; this will also require a resizing of the cell in the plane to maintain periodicity - see Figure 9), to allow us to include precisely four atoms per monolayer (as required for the minimal representation of 1/4 ML doping). We now have a situation where the *X*_TET _point in the new tetragonal BZ (see Figure 10) is no longer in the direction of the *X*_SC_ point in the simple cubic BZ, despite both *X* points being in the centre of a face of their BZ. Due to the rotation, what used to be the Δ_SC_ direction in the simple cubic BZ is now the Σ_TET_ direction (pointing towards *M* at the corner of the BZ in the *k*_*z *_= 0 plane) in the tetragonal BZ. The tetragonal CBM, while physically still the same as the CBM in the FCC or simple cubic BZ, is not represented in the same fashion (see Figure 11).

## Appendix 2

### Band folding in the *z* direction

Increasing the *z* dimension of the cell leads to successive folding points being introduced as the BZ shrinks along *k*_*z *_(see Appendix 1). This has the effect of shifting the conduction band minima in the ±* k*_*z*_ directions closer and closer to the Γ point (see Figure 8a) and making the band structure extremely dense when plotting along *k*_*z*_. This results in the value of the lowest unoccupied eigenstate at Γ being lowered as what were originally other sections of the band are successively mapped onto Γ, and after a sufficient number of folds, the value at Γ is indistinct from the original CBM value. The effects of this can be seen in Table 4, which describes increasingly elongated tetragonal cells of bulk Si. When we then plot the band structure in a different direction, e.g. along *k*_*x*_, the translation of the minima from ±* k*_*z *_onto the Γ point appear as a new band with twofold degeneracy. The degeneracy of the original band seems to drop from six- to fourfold, in line with the reduced symmetry (we only explicitly calculate one, and the other three occur due to symmetry considerations). This is half of the origin of the ‘Γbands’ (more details are presented in Appendix 3). Once the *k*_*z*_ valleys are sited at Γ, parabolic dispersion corresponding to the transverse kinetic energy terms is observed along *k*_*x*_ and *k*_*y*_, at least close to the band minimum (see Figure 11) - in contrast to the four ‘*δ*bands’ whose dispersion (again parabolic) is governed by the longitudinal kinetic energy terms. The different curvatures are related to the different effective masses (transverse, longitudinal) of the silicon CBM. It should be noted that the bands are still degenerate in energy at this stage - their minima (and range) occur at (over) the same energy (energies) even though their projections onto the *k*_*x*_ axis are different.

**Table 4:** Energy levels of tetragonal bulk Si structures

**Basis**	**Number of**	**Number of**	**LUMO**	**CBM**
**type**	**layers**	***k*-pts**	**at Γ**	**(at Δ**_**FCC**_**)**
		**in *k***_***z***_	**(eV)**	**(eV)**
PW	4	12	0.7517	
(vasp)	8	6	0.7517	
	16	3	0.6506	
	32	2	0.6170	
	40	1	0.6179	
	64	1	0.6137	
	80	1	0.6107	0.6102
DZP	40	1	0.6218	
(siesta)	60	1	0.6194	
	80	1	0.6154	
	120	1	0.6145	
	160	1	0.6151	0.6145
SZP	40	1	0.8392	
(siesta)	60	1	0.8349	
	80	1	0.8315	
	120	1	0.8311	
	160	1	0.8315	
	200	1	0.8310	0.8309

For details of the calculation parameters, see the ‘Methods’ section.

All methods considered in Table 4 show the LUMO at Γ (folded in along ±* k*_*z*_) approaching the CBM value as the amount of cladding increases; at 80 layers, the LUMO at Γ is within 1 meV of the CBM value. It is also of note that the PW indirect bandgap agrees well with the DZP value and less so with the SZP model. This is an indication that, although the behaviour of the LUMO with respect to the cell shape is well replicated, the SZP basis set is demonstrably incomplete. Conversely, pairwise comparisons between the PW and DZP results show agreement to within 5 meV.

It is important to distinguish effects indicating convergence with respect to cladding for doped cells (i.e. elimination of layer-layer interactions) from those mentioned previously derived from the shape and size of the supercell. Strictly, the convergence (with respect to the amount of encapsulating Si) of those results we wish to study in detail, such as the differences in energy between occupied levels in what was the bulk bandgap, provides the most appropriate measure of whether sufficient cladding has been applied.

## Appendix 3

### Valley splitting

Here, we discuss the origins of valley splitting, in the context of phosphorus donors in silicon. Following on from the discussion of Si band minima in Appendices 1 and 2, we have, via elongation of the supercell and consequent band folding, a situation where, instead of the sixfold degeneracy (due to the underlying symmetries of the Si crystal lattice), we see an apparent splitting of these states into two groups (6 → 2 + 4, or 2 Γ + 4 *δ *minima).

We now consider what happens in perfectly ordered *δ*-doped monolayers, as per the main text. Here, we break the underlying Si crystal lattice symmetries by including foreign elements in the lattice. By placing the donors regularly (according to the original Si lattice pattern) in one [001] monolayer, we reduce the symmetry of the system to tetragonal, with the odd dimension being transverse to the plane of donors. This dimension can be periodic (as in the supercells described earlier), infinite (as in the EMT model of Drumm et al. [40]) or extremely long on the atomic scale (as the experiments are).

Immediately, therefore, we expect the same apparent 2 + 4 breaking of the original sixfold degenerate conduction band minima. Of course, as we have introduced phosphorus (which has one more electron and one more proton than silicon), this next band (still actually sixfold degenerate in bulk silicon) will be occupied and will now be influenced by the new potential. The sub-bands interact differently with the potential, thanks to the different curvatures in their dispersion relations and drop by different amounts into the bandgap. As discussed in detail in Drumm et al. [40], the filling of these sub-bands is partial rather than complete (or absent) and is governed by both the energy of their minima and their respective effective masses. We now have an actual breaking of the sixfold degeneracy into a true 2 + 4 system.

If we still look closer, we might expect these lower degeneracies to spontaneously break - nature, after all, is said to abhor degeneracy. Indeed, this does occur, but for this special case of *δ*-doped Si:P, the effect is enhanced by the strong V-shaped potential about the monolayer due to the extra charge in the donor nuclei [40]. Consideration of odd and even solutions to the effective mass Schrödinger equation for this sub-band leads to their superposition(s) and subsequent energy difference. This is enhanced further in the Kohn-Sham formalism, as evidenced in previous sections. (The four *δ* minima also split but on a far-reduced scale not visible using current DFT techniques.) We thus expect, in the DFT picture, to see 6 →2 + 4→1 + 1 + 4 sub-band structure, namely the Γ_1_, Γ_2_ and *δ *bands. The valley splitting which is the main focus of this paper is the energy difference between the Γ_1_ and Γ_2_ band minima due to the superposition of solutions.

## Competing interests

The authors declare that they have no competing interests.

## Authors’ contributions

DWD, SPR, and LCLH conceived the study. Density functional theory calculations were carried out by DWD, AB, and MCP. All authors contributed to the discussion of results and drafting of the the final manuscript. All authors read and approved the final manuscript.
